# Direct Effects of Vitamin D Supplementation on Ultramarathon-Induced Changes in Kynurenine Metabolism

**DOI:** 10.3390/nu14214485

**Published:** 2022-10-25

**Authors:** Jan Mieszkowski, Paulina Brzezińska, Błażej Stankiewicz, Andrzej Kochanowicz, Bartłomiej Niespodziński, Joanna Reczkowicz, Tomasz Waldziński, Bartłomiej Kacprzak, Natalia Siuba-Jarosz, Miroslav Petr, Jędrzej Antosiewicz

**Affiliations:** 1 Department of Gymnastics and Dance, Gdansk University of Physical Education and Sport, Kazimierza Gorskiego 1, 80-336 Gdansk, Poland; 2 Faculty of Physical Education and Sport, Charles University, 162 52 Prague, Czech Republic; 3 Institute of Physical Education, Kazimierz Wielki University, Jana Karola Chodkiewicza 30, 85-064 Bydgoszcz, Poland; 4 Medical Department of Bioenergetics and Physiology of Exercise, Faculty of Health Sciences, Medical University of Gdansk, Dębinki 1, 80-211 Gdansk, Poland; 5 Faculty of Health Sciences, Łomża State University of Applied Science, Akademicka 14, 18-400 Lomza, Poland; 6 Faculty of Medical and Health Sciences, University of Humanities and Economics in Lodz, Sterlinga 26, 90-212 Lodz, Poland; 7 Orto Med Sport, 28 Pulku Strzelcow Kaniowskich 45, 90-640 Lodz, Poland

**Keywords:** kynurenine, ultramarathon, skeletal muscle damage, vitamin D

## Abstract

In humans, most free tryptophan is degraded via kynurenine pathways into kynurenines. Kynurenines modulate the immune system, central nervous system, and skeletal muscle bioenergetics. Consequently, kynurenine pathway metabolites (KPMs) have been studied in the context of exercise. However, the effect of vitamin D supplementation on exercise-induced changes in KPMs has not been investigated. Here, we analyzed the effect of a single high-dose vitamin D supplementation on KPMs and tryptophan levels in runners after an ultramarathon. In the study, 35 amateur runners were assigned into two groups: vitamin D supplementation group, administered 150,000 IU vitamin D in vegetable oil 24 h before the run (*n* = 16); and control (placebo) group (*n* = 19). Blood was collected for analysis 24 h before, immediately after, and 24 h after the run. Kynurenic, xanthurenic, quinolinic, and picolinic acids levels were significantly increased after the run in the control group, but the effect was blunted by vitamin D supplementation. Conversely, the decrease in serum tryptophan, tyrosine, and phenylalanine levels immediately after the run was more pronounced in the supplemented group than in the control. The 3-hydroxy-l-kynurenine levels were significantly increased in both groups after the run. We conclude that vitamin D supplementation affects ultramarathon-induced changes in tryptophan metabolism.

## 1. Introduction

Tryptophan (TRP) is an essential amino acid required for several biological processes, including protein, serotonin, and melatonin biosynthesis. Most of the serum TRP is degraded via kynurenine (KYN) pathways. The conversion of TRP to KYN is mediated by the enzymes TRP 2,3-dioxygenase (TDO) or indoleamine 2,3-dioxygenase (IDO). In skeletal muscle, the enzymatic activity of these two enzymes is low. Hence, during exercise, KYN is likely produced outside the muscle and then imported into it [1].

Within skeletal muscle and other tissues, KYN is metabolized into kynurenic acid (KYNA), anthranilic acid (AA), and xanthurenic acid (XANA), quinolinic acid (QA), 3-hydroxy-l-KYN (3-HK) and picolinic acid (PA), and an increase in KYN serum levels has been observed after different types of exercise [1,2]. On the other hand, one of the characteristics of adaptation to endurance training is the increased expression of KYN aminotransferases (KATs), which catalyze the conversion of KYN to KYNA [3]. The physiological meaning of these changes is not fully understood. For example, KYNA formation is considered to be a positive development as, contrary to KYN, KYNA does not penetrate the blood–brain barrier. Hence, the conversion of KYN to KYNA protects the central nervous system from the adverse effects of KYN [4]. In addition, it has been shown that glutamate formed in a KAT-catalyzed reaction enhanced the operation of the malate–aspartate shuttle, with an overall improvement in the skeletal muscle bioenergetics [5]. Conversely, the formation of QA can have some adverse effects as it is neurotoxic and can stimulate the formation of reactive oxygen species, which in turn activates the *N*-methyl-d-aspartate receptor, dysregulates glutamate release and uptake by neurons and astrocytes, and stimulates lipid peroxidation [6,7].

The TRP metabolism can be modified by inflammatory cytokines [3]. It has been shown that high cortisol and inflammatory cytokine levels lead to the suppression of hepatic TDO and the induction of IDO in immune cells [8,9,10]. Continuous endurance (among others, ultramarathon and marathon) effort-induced inflammation is well documented [11,12] and, hence, one could expect that exercise-induced inflammation would affect TRP metabolism. Several studies have demonstrated that exercise enhances the TRP metabolism, with elevated serum levels of KYNA, KYN, etc. [13]. However, the role of exercise-induced inflammation in these changes has not been evaluated in detail.

Recently, we have demonstrated that vitamin D supplementation reduces ultramarathon-induced inflammation [14]. The anti-inflammatory activity of vitamin D is well recognized, as its active form has been shown to repress the expression of proinflammatory cytokines [14]. Among its renowned functions, vitamin D has been much studied recently in relation to its response against viral infections (especially against COVID-19) and in relation to other metabolic pathways in the body that regulate body functioning [15,16].

Furthermore, it has been suggested that vitamin D represses tryptophan hydroxylase (TPH1), thus making TRP available for the KYN pathway [17]. Both KYN and KYNA enhance the differentiation of naive T cells to regulatory T cells, which suppress the inflammatory response [18,19]. Considering the above, in the current study, we evaluated the effect of a single high-dose vitamin D supplementation on ultramarathon-induced changes in TRP metabolism. We hypothesized that increased serum concentration of vitamin D by reducing exercise-induced inflammation will modify tryptophan metabolism and affect kynurenine metabolites.

## 2. Materials and Methods

### 2.1. Experimental Overview

The study was designed as a double-blind, randomized, controlled trial with parallel groups. Participants were randomly assigned to two groups: the supplementation group and the control group. The supplementation protocol involved the administration of a single high dose of vitamin D_3_ before the ultramarathon. During the initial site visit, data on the subject’s age, body composition, and height were collected. All runners were examined by a professional physician. A venous blood sample was obtained before the ultramarathon start, and immediately after and 24 h after the run, to evaluate the effect of a high dose of vitamin D on ultramarathon-induced changes in the KYN metabolism. Due to the fact that the size of the female population taking part in the ultramarathon run—and in the study—did not allow for the construction of the experimental group, the above study applies only to men.

All laboratory analyses were performed at the Gdansk University of Physical Education (Gdansk, Poland).

### 2.2. Participants

Thirty-five amateur male ultramarathon runners who took part in the Lower Silesian Mountain Run Festival Ultramarathon Race participated in the study. All participants had been informed about the study procedures prior to enrollment, but they were not aware of the study aims or the supplementation schedule. The minimal population sample size allowing for the appropriate power of the study of interactions between the effects was 28 subjects, as calculated using GPower ver. 3.1.9.2 software. The runners were randomly assigned to two groups: experimental (supplementation, S; *n* = 16; aged 42.40 ± 7.59 years) and control (placebo, C; *n* = 19; aged 39.48 ± 6.89 years), according to the eligibility criteria presented elsewhere [14].

The basic anthropological characteristics of the participants are presented elsewhere [14], and include training regime and load, maximum oxygen uptake capacity (VO_2_ max) based on the Cooper test, and relevant information related to the time period preceding the research and sport performance.

According to the medical declarations and diagnostics carried out prior to the qualification for the study, none of the runners had a history of known diseases or reported any intake of medication due to illnesses during the 6 months before the experiment. On the measurement days, each participant was asked to adopt a similar eating pattern based on a randomized diet adjusted for their sex, age, work, and physical activity. All study protocols have been accepted by the Bioethics Committee for Clinical Research of the Collegium Medicum University of Nicolaus Copernicus (decision number KB-124/2017) and conducted according to the Declaration of Helsinki. The study is a registered clinical trial (NCT03417700).

Written informed consent was obtained from all study participants prior to the study, who were also informed about the possibility of the withdrawal of consent at any time and for any reason.

### 2.3. Vitamin D Supplementation

Vitamin D supplementation involved the administration of a single high dose of vitamin D_3_ (150,000 IU) to all participants in the experimental group 24 h before the start of the ultramarathon. The control population received a placebo solution whose taste (anise), color, and consistency matched those of the vitamin D solution (pure vegetable oil solution). The participants and researchers had no knowledge of the groups and differences in the supplementation procedures.

### 2.4. Sample Collection, and Measurements of Vitamin D and KYN Metabolite Levels

Blood samples (9 mL) were collected three times: 24 h before the run, immediately after the run (up to 5 min after the run), and 24 h after the run. Venous blood samples were collected into Sarstedt S-Monovette tubes (S-Monovette^®^ Sarstedt AG&Co, Nümbrecht, Germany) without anticoagulant for serum separation (the tubes contained a coagulation accelerator). The serum was separated using standard laboratory procedures, aliquoted, and frozen at −80 °C until further analysis [14]. Sample preparation was based on serum protein precipitation and derivatization. 4-(4′-Dimethylaminophenyl)-1,2,4-triazoline-3,5-dione was used as the derivatization agent and was synthesized at Masdiag Laboratory (Warsaw, Poland). Quantitative analysis of vitamin D metabolites was performed using liquid chromatography coupled with tandem mass spectrometry (LC-MS/MS) (QTRAP^®^ 4500 (Sciex) coupled with ExionLC HPLC system) according to Rola et al., 2020, with minor changes. KYN metabolites were also analyzed using LC-MS/MS, according to Midttun et al., 2009.

Serum samples were analyzed in the positive ion mode, using electrospray ionization. The raw data were collected using LabSolutions LCGC. LabSolutions LCGC was also used to process and quantify the collected data. Mobile phases were prepared using acetonitrile (Honeywell, Sigma-Aldrich, Gillingham, Dorset, UK), water (POCh S.A., Gliwice, Poland), and formic acid (Merck KGaA, Darmstadt, Germany). All solvents were LC-MS grade.

The following vitamin D metabolites were analyzed: 25(OH)D_3_ and 24,25(OH)_2_D_3_ (after a correction for changes in plasma volume). The following KYN metabolites were analyzed: 3-HK, KYN, KYNA, PA, QA, TRP, and XANA.

### 2.5. Ultramarathon Run

On the day following the first blood sample collection, physical examination, and supplementation protocol, all study participants took part in the Lower Silesian Mountain Run Festival (19 July 2018). The start and finish points were in the town of Lądek Zdrój (Lower Silesian Voivodeship, Poland). The running festival took place in the Kłodzko Land (latitude of 50° N), and consisted of seven mountain trails, with the maximum course length of 240 km, maximum altitude of approximately 1425 m MSL, and minimum altitude of approximately 261 m MSL. The entire altitude range was approximately 1164 m, and the total ascent and descent was 7670 m. The run started at 18:00 h. The temperature during the run varied from 18 °C at the start point to 4 °C on the top of the Śnieżnik Mountain. The sky was overcast for most of the run.

### 2.6. Statistical Analysis

Descriptive statistics included mean ± standard deviation (SD) for all measured variables. Two-way analysis of variance (ANOVA) with repeated measures (2 × 3) was performed to investigate the effect of the ultramarathon run (*UM*: 24 h before, immediately after, and 24 h after the run) on KYN metabolites and physical characteristics in relation to vitamin D supplementation (*group*: supplemented, S; control, C). In case of significant interaction, Tukey’s post hoc test was performed to assess differences in specific subgroups. The effect size was determined by eta-squared statistics (η^2^). Values equal to or more than 0.01, 0.06, and 0.14 indicated a small, moderate, and large effect, respectively. All calculations and graphics preparation were carried out using Statistica 12 software (StatSoft, Tulsa, OK, USA). Differences were considered statistically significant when *p* ≤ 0.05.

## 3. Results

The analysis of serum levels of vitamin D metabolites revealed their significant increase immediately after, and 24 h after, ultramarathons in both study groups. However, considering the hematocrit-adjusted data, the changes noted in the supplementation group immediately after the run (25(OH)D_3_, Δ = 46.67 ± 14.87 ng/mL; 24,25(OH)_2_D_3_, Δ = 3.34 ± 1.48 ng/mL) were significantly greater than those in the control group (25(OH)D_3_, Δ = 33.06 ± 8.08 ng/mL, *p* < 0.01; 24,25(OH)_2_D_3_, Δ = 2.51 ± 0.94 ng/mL, *p* < 0.05). The initial serum levels of 25(OH)D_3_ and 24,25(OH)_2_D_3_ were comparable in both groups.

**Figure 1:**
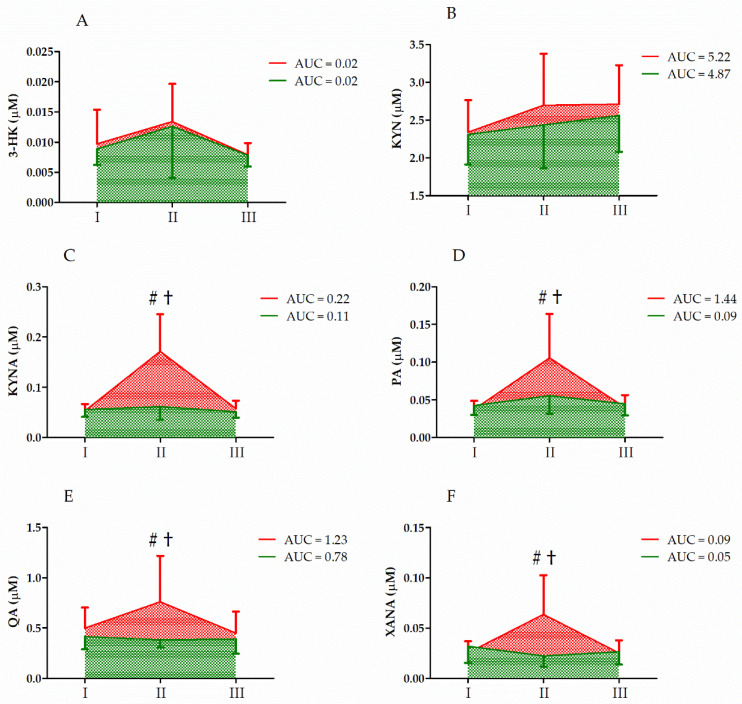
Changes in serum KYN metabolite levels after the ultramarathon in runners who received a single high dose of vitamin D (supplemented group, green) and runners who received the placebo (control group, red). Sampling: I, 24 h before the run; II, immediately after the run; and III, 24 h after the run. (**A**) 3-HK, 3-hydroxy-l-kynurenine; (**B**) KYN, kynurenine; (**C**) KYNA, kynurenic acid; (**D**) PA, picolinic acid; (**E**) QA, quinolinic acid; (**F**) XANA, xanthurenic acid. †, Significant difference vs. 24 h before and 24 after the run; #, significant difference vs. supplemented group immediately after the run. The significance threshold was set at *p* < 0.01.

The analysis and changes in KYN metabolite levels induced by the ultramarathon run are presented in Table 1 and Figure 1, respectively. A significant effect of *UM* on all analyzed KYN metabolites was detected. A significant increase in the serum levels of 3-HK (129.97%), KYN (11.20%), KYNA (137.03%), PA (115.38%), QA (29.58%), and XANA (64.28%) was observed immediately after the run in placebo. In addition, two-way ANOVA revealed a significant effect of the *group* factor and a significant interaction of the *group* factor and *UM* for KYNA, PA, QA, and XANA. The levels of KYNA, PA, QA, and XANA immediately after the ultramarathon were significantly increased (vs. 24 h before the run, *p* < 0.01, post hoc analysis) only in the runners in the C group. The serum levels of the mentioned markers immediately after the run were significantly different between the groups (Figure 1).

**Figure 2:**
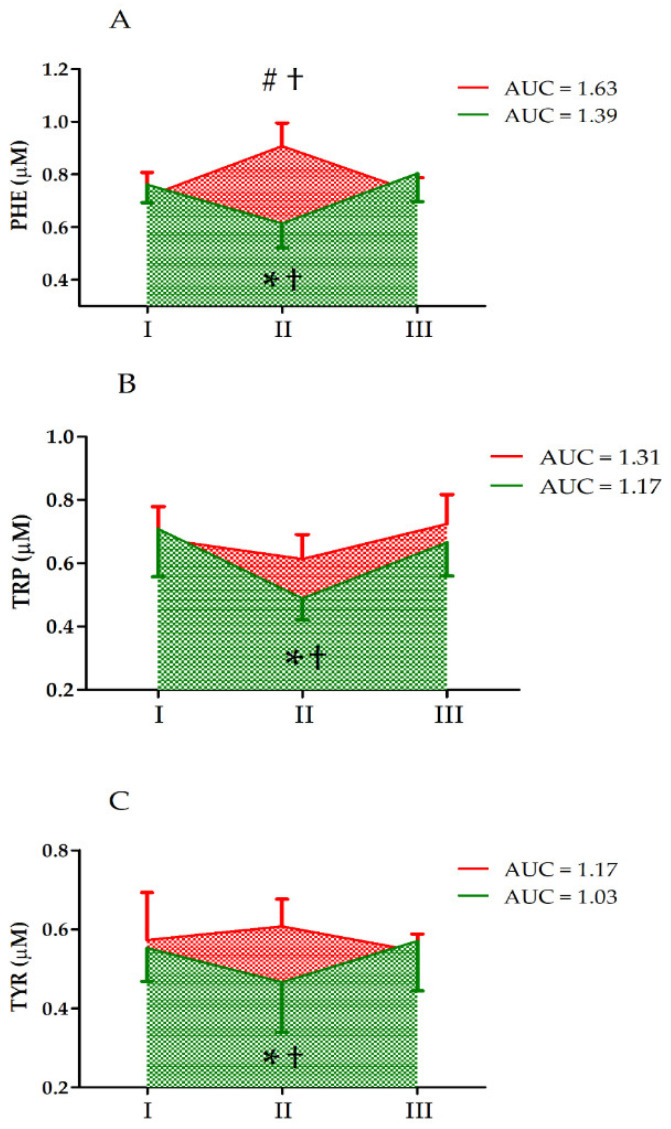
Changes in serum levels of (**A**) phenylalanine (PHE), (**B**) tryptophan (TRP), and (**C**) tyrosine (TYR) after the ultramarathon in runners who received a single high dose of vitamin D (supplemented group, green) and runners who received the placebo (control group, red). Sampling: I, 24 h before the run; II, immediately after the run; and III, 24 h after the run. †, Significant difference vs. 24 h before and 24 after the run; #, significant difference vs. supplemented group immediately after the run; *, significant difference vs. control group immediately after the run. The significance level was set at *p* < 0.01.

Further, two-way ANOVA indicated a significant interaction of the *group* and *UM* factors for PHE, TRP, and TYR (Table 2). We detected a significant decrease in the PHE, TRP, and TYR levels in the S group immediately after the ultramarathon (post hoc analysis). Further, there were significant differences between the groups and a significant increase in PHE levels in group C immediately after the ultramarathon (Figure 2).

**Figure 3:**
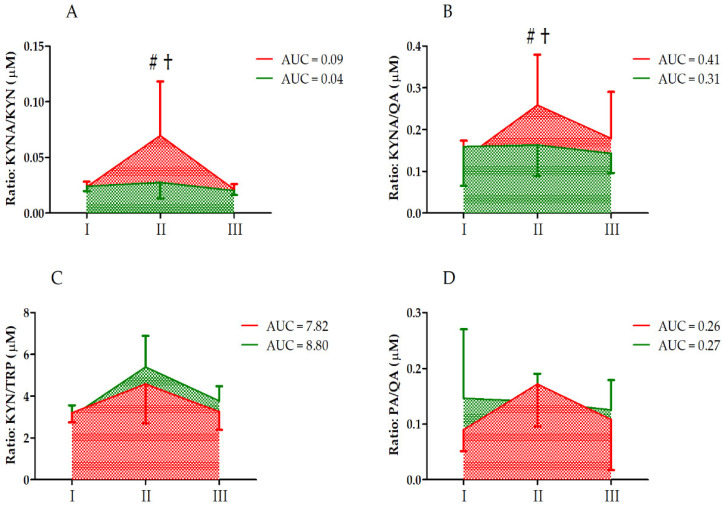
Changes in KYN metabolite and TRP ratios after the ultramarathon in runners who received a single high dose of vitamin D (supplemented group , green) and runners who received the placebo (control group, red). Sampling: I, 24 h before the run; II, immediately after the run; and III, 24 h after the run. (**A**) KYNA/KYN, kynurenic acid to kynurenine ratio; (**B**) KYNA/QA, kynurenic acid to quinolinic acid ratio; (**C**) KYN/TRP, kynurenine to tryptophan ratio; (**D**) PA/QA, picolinic acid to quinolinic acid ratio. †, Significant difference vs. 24 h before and 24 after the run; #, significant difference vs. supplemented group immediately after the run. The significance level was set at *p* < 0.01.

Further, analysis of the KYNA/KYN and KYN/TRP ratios revealed a significant effect of the UM factor on these ratios (Table 3). However, significant interaction of the group and UM factors was noted only for the KYNA/KYN ratio. The KYNA/KYN ratio was significantly increased in group C immediately after the ultramarathon, with a significant difference between the groups (post hoc analysis, Figure 3). According to two-way ANOVA, the UM and group factors did not significantly affect the KYNA/QA and PA/QA ratios (Table 3).

## 4. Discussion

In the present study, we demonstrated that a single high dose of vitamin D significantly influences the TRP metabolism after an ultramarathon run and changes KYNA, QA, PA, and XANA concentrations. The single high dose can influence exhaustion and possibly protect from known adverse effects of some TRP metabolites on the central nervous system and circulation [20].

In vitamin-D-supplemented athletes, the decrease in serum TRP levels was more pronounced that that in the control group (Figure 2), but the increase in the levels of TRP metabolites KYNA, XANA, QA, and PA was blunted compared to that in the control group (Figure 3). An increase in circulating KYNA levels and a decrease in circulating TRP and KYN levels following acute endurance exercise have been reported in several studies [17,21,22]. This could be explained by the observation that endurance training induces the expression of KATs [23]. Specifically, in trained athletes, KYN is efficiently converted to KYNA, with a concomitant decrease in serum TRP levels [1]. The control group data obtained in the current study confirmed earlier observations on TRP metabolites [24]. Moreover, the higher increase in KYNA/KYN in control groups indicates that vitamin D somehow augments reactions catalyzed by KAT.

The effects of vitamin D supplementation on TRP metabolism are puzzling. First, in the supplementation group, the decrease in serum TRP levels after the run are more pronounced than that in the control group (Figure 2). During exercise, the serum TRP is taken up by different tissues and metabolized, and hence, its concentration should decrease. Typically, approximately 3–3.6 g is released and incorporated each day. Conversely, during exercise (e.g., ultramarathon), more skeletal muscle proteins undergo proteolytic degradation than are synthesized, and hence, more amino acids are exported into the blood. Further, it has been shown that vitamin D supplementation reduces atrogin-1 levels in human skeletal muscle [25]. Atrogin-1 stimulates skeletal muscle atrophy and its expression increases following endurance exercise [26]. Hence, it is possible that skeletal muscle proteolysis is partially inhibited in athletes supplemented with a high dose of vitamin D. Consequently, the more pronounced decrease in serum TRP levels in the supplementation group (vs. that in the control group) may indicate that relatively lower amounts of amino acids are liberated from skeletal muscle or other tissue during exercise in that group. Conversely, as the substrate (TRP) concentration decreases, the formation of the metabolites could also decrease. Further, an increase in 3-HK and KYN levels in the S group indicates that an ultramarathon stimulates the TRP metabolism in athletes (Figure 1). In addition, changes in the serum levels of the aromatic amino acids TYR and PHE in the control group were distinct from those in the vitamin-D-supplemented group (Figure 2). These two amino acids are not degraded by skeletal muscle, and could therefore be used as indicators of the balance between protein synthesis and degradation. We did not determine their levels in the skeletal muscle; however, a significant increase in the serum PHE levels in the control group and their decrease in the vitamin-D-supplemented group indicates higher proteolysis in the former.

We have previously demonstrated that high doses of vitamin D effectively reduce ultramarathon-induced inflammation [14]. Inflammatory stimuli augment the TRP metabolism and KYN formation. Interferon gamma, tumor necrosis factor, and some other proinflammatory cytokines enhance IDO protein activity and TRP breakdown [27]. Further, exercise-induced changes in KYN levels correlate with the changes in inflammatory cytokine levels, suggesting that these processes are functionally linked [21]. Hence, the data presented herein allow us to speculate that a decrease in inflammation mediated by vitamin D supplementation was partially responsible for a decrease in KYN levels after the ultramarathon run.

A small portion of serum TRP is metabolized in the central nervous system into serotonin [19]. It has been shown that an increase in the plasma levels of free TRP can result in an increased passage of TRP into the brain via the blood–brain barrier, which augments the formation of serotonin in the brain and exercise-induced fatigue [28,29]. Conversely, in an animal rat model, a low-TRP diet results in improved endurance performance [30]. Hence, the observed pronounced decrease in serum TRP after the run in vitamin-D-supplemented athletes could be considered as a positive change that delays exhaustion.

The main limitation of this study is that activity of enzymes involved in TRP metabolism has not been measured in skeletal muscle and white blood cells.

Overall, we have shown that vitamin D supplementation which increases its blood concentration significantly reduced the ultramarathon-induced increase in KYNA, QA, PA, and XANA. These findings implicate that vitamin D supplementation can significantly modify TRP metabolism during exercise, can influence exhaustion, and possibly protect from known adverse effects of some TRP metabolites on the central nervous system and circulation.

## Figures and Tables

**Table 1:** Two-way ANOVA (2 groups × 3 repeated measures) of changes in serum KYN metabolite levels induced by ultramarathon run.

Variable	Effect	F	Df	*p*	Effect Size (η^2^)	Post Hoc Outcome
3-HK	GR UM GR × UM	0.18 6.00 0.04	1, 33 2, 66 2, 66	0.67 <0.01 ** 0.96	0.01 0.16 <0.01	II > I, III
KYN	GR UM GR × UM	1.01 7.66 1.36	1, 33 2, 66 2, 66	0.32 <0.01 ** 0.27	0.03 0.19 0.04	II, III > I
KYNA	GR UM GR × UM	9.47 11.87 8.81	1, 33 2, 66 2, 66	<0.01 ** <0.01 ** <0.01 **	0.23 0.27 0.22	S < C II > I, III CII > CI, CIII CII > SII
PA	GR UM GR × UM	4.99 22.39 13.31	1, 33 2, 66 2, 66	0.03 * <0.01 ** <0.01 **	0.13 0.41 0.25	S < C II > I, III CII > CI, CIII CII > SII
QA	GR UM GR × UM	4.46 6.50 8.51	1, 33 2, 66 2, 66	<0.01 ** <0.01 ** <0.01 **	0.12 0.17 0.21	S < C II > I, III CII > CI, CIII CII > SII
XANA	GR UM GR × UM	4.22 4.94 10.25	1, 33 2, 66 2, 66	0.05 * 0.01 * <0.01 **	0.15 0.18 0.31	S < C II > I, III CII > CI, CIII

Note: Markers: 3-HK, 3-hydroxy-l-kynurenine; KYN, kynurenine; KYNA, kynurenic acid; PA, picolinic acid; QA, quinolinic acid; TRP, tryptophan; XANA, xanthurenic acid. Study design: GR, group effect; S, runners who received a single high dose of vitamin D; C, runners who received the placebo (control group); UM, ultramarathon run effect; I, 24 h before the run; II, immediately after the run; and III, 24 h after the run. Significant difference detected at * *p* ≤ 0.05 or ** *p* ≤ 0.01.

**Table 2:** Two-way ANOVA (2 groups × 3 repeated measures) of changes in serum levels of phenylalanine (PHE), tryptophan (TRP), and tyrosine (TYR) induced by ultramarathon run.

Variable	Effect	F	Df	*p*	Effect Size (η^2^)	Post Hoc Outcome
PHE	GR UM GR × UM	2.85 0.54 27.26	1, 33 2, 66 2, 66	0.11 0.58 <0.01 **	0.19 0.04 0.69	CII > CI, CIII SII < SI, SIII CII > SII
TRP	GR UM GR × UM	0.53 13.45 5.04	1, 33 2, 66 2, 66	0.47 <0.01 ** 0.01 *	0.04 0.52 0.29	II < I, III SII < SI, SIII CII > SII
TYR	GR UM GR × UM	1.03 0.34 3.47	1, 33 2, 66 2, 66	0.32 0.71 0.04 *	0.07 0.02 0.22	SII < SI, SIII CII > SII

Study design: GR, group; S, runners who received a single high dose of vitamin D; C, runners who received the placebo (control group); UM, ultramarathon; I, 24 h before the run; II, immediately after the run; and III, 24 h after the run. Significant difference detected at * *p* ≤ 0.05 or ** *p* ≤ 0.01.

**Table 3:** Two-way ANOVA (2 groups × 3 repeated measures) of changes in KYN metabolite and TRP ratios induced by ultramarathon run.

Variable	Effect	F	Df	*p*	Effect Size (η^2^)	Post Hoc Outcome
KYNA/KYN	GR UM GR × UM	8.28 12.60 7.36	1, 33 2, 66 2, 66	<0.01 ** <0.01 ** <0.01 **	0.21 0.28 0.19	S < C II > I, III CII > CI, CIII CII > SII
KYNA/QA	GR UM GR × UM	2.36 2.60 2.27	1, 33 2, 66 2, 66	0.13 0.08 0.11	0.06 0.07 0.06	
KYN/TRP	GR UM GR × UM	0.73 17.18 1.15	1, 33 2, 66 2, 66	0.40 <0.01 ** 0.33	0.05 0.58 0.08	II > I, III
PA/QA	GR UM GR × UM	0.24 2.15 2.40	1, 33 2, 66 2, 66	0.62 0.12 0.09	0.01 0.06 0.07	

Note: KYNA/KYN, kynurenic acid to kynurenine ratio; KYNA/QA, kynurenic acid to quinolinic acid ratio; KYN/TRP, kynurenine to tryptophan ratio; PA/QA, picolinic acid to quinolinic acid ratio. Study design: GR, group; S, runners who received a single high dose of vitamin D; C, runners who received the placebo (control group); UM, ultramarathon. Sampling: I, 24 h before the run; II, immediately after the run; and III, 24 h after the run. Significant difference detected at * *p* ≤ 0.05 or ** *p* ≤ 0.01.

## Data Availability

Datasets analyzed during the current study will be available at the end of the project of which they are part (Grant from the National Science Centre, Poland—number 2020/37/B/NZ7/01794).

## References

[1] Joisten N., Kummerhoff F., Koliamitra C., Schenk A., Walzik D., Hardt L., Knoop A., Thevis M., Kiesl D., Metcalfe A.J. (2020). Exercise and the Kynurenine pathway: Current state of knowledge and results from a randomized cross-over study comparing acute effects of endurance and resistance training. Exerc. Immunol. Rev..

[2] Kurgan S., Onder C., Balci N., Akdogan N., Altingoz S.M., Serdar M.A., Gunhan M. (2022). Influence of periodontal inflammation on tryptophan-kynurenine metabolism: A cross-sectional study. Clin. Oral Investig..

[3] Wang Q., Liu D., Song P., Zou M.H. (2015). Tryptophan-kynurenine pathway is dysregulated in inflammation, and immune activation. Front. Biosci. A J. Virtual Libr..

[4] Agudelo L.Z., Femenia T., Orhan F., Porsmyr-Palmertz M., Goiny M., Martinez-Redondo V., Correia J.C., Izadi M., Bhat M., Schuppe-Koistinen I. (2014). Skeletal muscle PGC-1alpha1 modulates kynurenine metabolism and mediates resilience to stress-induced depression. Cell.

[5] Agudelo L.Z., Ferreira D.M.S., Dadvar S., Cervenka I., Ketscher L., Izadi M., Zhengye L., Furrer R., Handschin C., Venckunas T. (2019). Skeletal muscle PGC-1alpha1 reroutes kynurenine metabolism to increase energy efficiency and fatigue-resistance. Nat. Commun..

[6] Vecsei L., Szalardy L., Fulop F., Toldi J. (2013). Kynurenines in the CNS: Recent advances and new questions. Nat. Rev. Drug Discov..

[7] Guillemin G.J. (2012). Quinolinic acid, the inescapable neurotoxin. FEBS J..

[8] Campbell B.M., Charych E., Lee A.W., Moller T. (2014). Kynurenines in CNS disease: Regulation by inflammatory cytokines. Front. Neurosci..

[9] Martin K.S., Azzolini M., Lira Ruas J. (2020). The kynurenine connection: How exercise shifts muscle tryptophan metabolism and affects energy homeostasis, the immune system, and the brain. Am. J. Physiol. Cell Physiol..

[10] Savitz J. (2020). The kynurenine pathway: A finger in every pie. Mol. Psychiatry.

[11] Alves M.D.J., Silva D.D.S., Pereira E.V.M., Pereira D.D., de Sousa Fernandes M.S., Santos D.F.C., Oliveira D.P.M., Vieira-Souza L.M., Aidar F.J., de Souza R.F. (2022). Changes in Cytokines Concentration Following Long-Distance Running: A Systematic Review and Meta-Analysis. Front. Physiol..

[12] Rudarli Nalcakan G., Onur E., Oran A., Varol S.R. (2021). Comparison of sprint interval and continuous endurance training on oxidative stress and antioxidant adaptations in young healthy adults. Balt. J. Health Phys. Act..

[13] Petrus P., Cervantes M., Samad M., Sato T., Chao A., Sato S., Koronowski K.B., Park G., Alam Y., Mejhert N. (2022). Tryptophan metabolism is a physiological integrator regulating circadian rhythms. Mol. Metab..

[14] Mieszkowski J., Borkowska A., Stankiewicz B., Kochanowicz A., Niespodzinski B., Surmiak M., Waldzinski T., Rola R., Petr M., Antosiewicz J. (2021). Single High-Dose Vitamin D Supplementation as an Approach for Reducing Ultramarathon-Induced Inflammation: A Double-Blind Randomized Controlled Trial. Nutrients.

[15] Abushamma A.A. (2022). The Effects of Vitamin D Supplementation on Athletic Performance and Injury Prevention. J. Sport. Med. Allied Health Sci. Off. J. Ohio Athl. Train. Assoc..

[16] Baciur P., Chmura A., Skowrońska K., Białas F., Kondel K. (2022). The role of Vitamin D in the prevention and treatment of inflammatory skin diseases—Atopic dermatitis and psoriasis—Literature review. J. Educ. Health Sport.

[17] Sabir M.S., Haussler M.R., Mallick S., Kaneko I., Lucas D.A., Haussler C.A., Whitfield G.K., Jurutka P.W. (2018). Optimal vitamin D spurs serotonin: 1, 25-dihydroxyvitamin D represses serotonin reuptake transport (SERT) and degradation (MAO-A) gene expression in cultured rat serotonergic neuronal cell lines. Genes Nutr..

[18] Weinhold M., Shimabukuro-Vornhagen A., Franke A., Theurich S., Wahl P., Hallek M., Schmidt A., Schinkothe T., Mester J., von Bergwelt-Baildon M. (2016). Physical exercise modulates the homeostasis of human regulatory T cells. J. Allergy Clin. Immunol..

[19] Patrick R.P., Ames B.N. (2014). Vitamin D hormone regulates serotonin synthesis. Part 1: Relevance for autism. FASEB J..

[20] Obara-Michlewska M. (2022). The tryptophan metabolism, kynurenine pathway and oxidative stress—Implications for glioma pathobiology. Neurochem. Int..

[21] Isung J., Granqvist M., Trepci A., Huang J., Schwieler L., Kierkegaard M., Erhardt S., Jokinen J., Piehl F. (2021). Differential effects on blood and cerebrospinal fluid immune protein markers and kynurenine pathway metabolites from aerobic physical exercise in healthy subjects. Sci. Rep..

[22] Puigarnau S., Fernàndez A., Obis E., Jové M., Castañer M., Pamplona R., Portero-Otin M., Camerino O. (2022). Metabolomics reveals that fittest trail runners show a better adaptation of bioenergetic pathways. J. Sci. Med. Sport.

[23] Schlittler M., Goiny M., Agudelo L.Z., Venckunas T., Brazaitis M., Skurvydas A., Kamandulis S., Ruas J.L., Erhardt S., Westerblad H. (2016). Endurance exercise increases skeletal muscle kynurenine aminotransferases and plasma kynurenic acid in humans. Am. J. Physiol. Cell Physiol..

[24] Mudry J.M., Alm P.S., Erhardt S., Goiny M., Fritz T., Caidahl K., Zierath J.R., Krook A., Wallberg-Henriksson H. (2016). Direct effects of exercise on kynurenine metabolism in people with normal glucose tolerance or type 2 diabetes. Diabetes Metab. Res. Rev..

[25] Dzik K.P., Skrobot W., Kaczor K.B., Flis D.J., Karnia M.J., Libionka W., Antosiewicz J., Kloc W., Kaczor J.J. (2019). Vitamin D Deficiency Is Associated with Muscle Atrophy and Reduced Mitochondrial Function in Patients with Chronic Low Back Pain. Oxidative Med. Cell. Longev..

[26] Louis E., Raue U., Yang Y., Jemiolo B., Trappe S. (2007). Time course of proteolytic, cytokine, and myostatin gene expression after acute exercise in human skeletal muscle. J. Appl. Physiol..

[27] Shirey K.A., Jung J.Y., Maeder G.S., Carlin J.M. (2006). Upregulation of IFN-gamma receptor expression by proinflammatory cytokines influences IDO activation in epithelial cells. J. Interf. Cytokine Res..

[28] Pardridge W.M. (1998). Blood-brain barrier carrier-mediated transport and brain metabolism of amino acids. Neurochem. Res..

[29] Cordeiro L.M.S., Rabelo P.C.R., Moraes M.M., Teixeira-Coelho F., Coimbra C.C., Wanner S.P., Soares D.D. (2017). Physical exercise-induced fatigue: The role of serotonergic and dopaminergic systems. Braz. J. Med. Biol. Res..

[30] Yamamoto T., Newsholme E.A. (2003). The effect of tryptophan deficiency in the brain on rat fatigue levels: A rat model of fatigue reduction. Adv. Exp. Med. Biol..

